# Frequency of Blood Transfusion in Percutaneous Nephrolithotomy

**DOI:** 10.7759/cureus.11086

**Published:** 2020-10-21

**Authors:** Sami Ullah, Sikandar Ali, Sundas Karimi, Umar Farooque, Manzoor Hussain, Faisal Qureshi, SM Ismail Shah, Anoshia Afzal, Abubakar Tauseef, Muhammad Umair Khan

**Affiliations:** 1 Urology, Pakistan Navy Ship Shifa Hospital, Karachi, PAK; 2 Urology, Sindh Institute of Urology and Transplantation, Karachi, PAK; 3 General Surgery, Combined Military Hospital, Karachi, PAK; 4 Neurology, Dow University of Health Sciences, Karachi, PAK; 5 Internal Medicine, Dow University of Health Sciences, Karachi, PAK; 6 Internal Medicine, Ziauddin Medical College, Karachi, PAK; 7 Pathology, University of Oklahoma Health Sciences Center, Oklahoma City, USA; 8 Internal Medicine, Creighton University, Omaha, USA; 9 Internal Medicine, Services Institute of Medical Services, Lahore, PAK

**Keywords:** percutaneous nephrolithotomy, nephrolithiasis, kidney stone, staghorn stone, bleeding risk, blood transfusion, frequency, humans, gender, operative time

## Abstract

Introduction

Percutaneous nephrolithotomy (PNL) has replaced open surgery for the treatment of kidney stones due to its less invasive nature. Bleeding still occurs due to renal vascular injuries, dependent upon the access route of the procedure. Several other factors are also related to the increased risk of bleeding. This study aims to find the association between blood transfusion and other factors such as age, gender, body mass index (BMI), size of the stone, operative time, preoperative hemoglobin (Hb) level, stone surface area, hypertension, and diabetes mellitus.

Materials and methods

This was a descriptive cross-sectional study conducted over a period of six months between November 2019 and April 2020 at a tertiary care hospital in Karachi, Pakistan. The sample size of 131 patients was calculated using open-source epidemiological software (Open-Epi). Inclusion criteria included patients from both genders and ages between 26 and 70 years. Patients ≤25 years, having a liver disease or bleeding disorders, or refusing to participate in the study, were excluded. Laboratory data included preoperative routine complete blood count, serum creatinine (normal 0.5-1.5 mg/dL), platelet count, bleeding and coagulation profile, and urine culture. All patients also underwent renal ultrasound scans. Treatment was postponed until a negative urine culture was obtained from patients with a positive urine culture.

Results

The mean age of the patients was 42.4 ± 15.65 years. One third (29.8%) of the patients were females. The stone size was 850 ± 121.43 mm², the mean operative time of the procedure was 125.76 ± 53.4 minutes, and the mean number of cell packs transfused was 1.10 ± 0.31 units. Blood transfusion was done in 24 (18.3%) of the patients. Gender, diabetes mellitus, stone size, preoperative Hb level, and operative time were significantly related to blood transfusion.

Conclusions

Increased bleeding risk while performing PNL has been associated with many factors such as operating time, the gender of the patients, and stone size. Therefore, these factors should be controlled for the procedure to decrease the risk of bleeding and the need for blood transfusion. Furthermore, the kidney vasculature should not be compromised while performing the procedure.

## Introduction

In percutaneous nephrolithotomy (PNL), the percutaneous technique of insertion of a small-caliber nephrostomy tube is used to access the kidney and drain an obstructed renal unit. Ever since its discovery, the percutaneous removal of kidney stone has replaced open techniques and is now being used with extracorporeal shockwave lithotripsy (SWL) in the clinical setting [1]. These newer techniques are far less invasive than open surgery. The positions for the procedure include the prone, supine, and flank positions, each having their limitations. Access to the renal pelvis through the posterior calyx is known to be the best route [2]. Access should be made as such to avoid vascular injuries, such as injuries to segmental and interlobar arteries [3]. Bleeding is a common complication of PNL. Surrounding organs can also get injured due to puncture causing pleural or colonic injury [4].

Many factors affect renal hemorrhages, such as the operative time, upper calyceal access, multiple access sites, method of tract dilatation, number of tracts, size and location of stones, staghorn calculi, diabetes mellitus in patients, and the experience of the surgeon [5]. Bleeding can be managed conservatively while some patients might require an invasive procedure. The blood transfusion rate has been reported to be between 11% and 23% [6]. At the same time, kidney stones can be infected, renal dysfunction can occur, operation time can be prolonged due to the volume of the stones, and eventually, blood loss can occur. Some of these factors need to be correlated with blood transfusion to determine how these factors might affect blood loss and the need for blood transfusion. The experience of the surgeon can determine the proper execution of the surgery. Yet complications such as sepsis, hypothermia, stricture formation, and mortality can occur.

Guidelines of the American Association of Urology (AUA) describe the use of PNL as the first-line for large kidney stones [7]. This prospective study aims to identify the frequency of blood transfusion during PNL and its relation to other factors such as age, gender, body mass index (BMI), size of the stone, operative time, preoperative hemoglobin (Hb) level, stone surface area, hypertension, and diabetes mellitus. Blood loss and blood transfusion during PNL can contribute to the burden on healthcare resources. The results of this study can be used for the better outcome of these patients. It will also provide baseline data that will add to the local and international studies.

## Materials and methods

Study design and sampling

This descriptive cross-sectional study was conducted from November 2019 to April 2020 for a duration of six months in the urology department of a tertiary care hospital in Karachi, Pakistan. A sample size (n) of 131 patients was calculated using open-source epidemiological software (Open-Epi). The expected prevalence was set as 14.2% at a 95% level of confidence and a 5% margin of error. The inclusion criteria included kidney stone patients aged 26-70 years of either gender. Patients aged ≤25 years, having a chronic liver disease or bleeding disorders as per their record, and refusing to participate in the study were excluded.

Data collection

Patients who fulfilled the inclusion criteria were included in the study and written informed consent was taken. Laboratory data included preoperative routine complete blood count, serum creatinine (normal 0.5-1.5 mg/dL), platelet count, bleeding and coagulation profile, and urine culture. Renal ultrasound scans were performed on all patients. Treatment was suspended until a negative urine culture was achieved from patients with a positive urine culture. Socioeconomic status was defined as lower class for patients with monthly income <10,000 rupees(Rs)/month, middle class for 10,000 - 30,000 Rs/month, and upper class for >30,000 Rs/month.

All PNL procedures were performed with patients in a prone position and under general anesthesia. The experience of the surgeon was at least three years. The entrance into the collecting system was confirmed on the removal of the stylet of the needle. A 0.038-inch J-tip wire was inserted and access was dilated. The tracts were dilated up to 30F in adults. The number of tracts was made according to the stone volume and dimensions. In all cases, pneumatic lithotripsy was used. Either 14F reentry or 8F pigtail nephrostomy tubes were inserted after completion. No hemostatic agent was used and the nephrostomy tube was clamped overnight and subsequently removed. The stone burden was calculated in mm² by the product of the longest dimension and the one perpendicular to it. Postoperative hemoglobin (Hb) and hematocrit were measured. Blood loss was calculated using the following formula: (preoperative blood Hb - postoperative Hb) + (number of units transfused x 1 g/dL per unit transfused) [8].

Data analysis

Data were entered and analyzed through the Statistical Package for Social Sciences (SPSS) version 23.0 (IBM Corp., Armonk, NY). Mean and the standard deviation was calculated for quantitative variables including age, BMI, serum creatinine level, size of the stone, operative time, number of cell packs, preoperative Hb level, decrease in Hb level, and stone surface area. Frequencies and percentages were calculated for qualitative variables such as hypertension, diabetes mellitus, preoperative urinary tract infection (UTI), gender, staghorn stone, chronic renal failure, socioeconomic status, number of tracts, access calyx, dilation method, side of puncture, and outcome (i.e., blood transfusion). Effect modifiers such as age, gender, socioeconomic status, BMI, hypertension, diabetes mellitus, size of the stone, stone surface area, preoperative Hb level, operative time, preoperative UTI, chronic renal failure, staghorn stone, number of tracts, access calyx, dilation method, and side of puncture were controlled through stratification. The post-stratification chi-square test was applied by taking p-value ≤0.05 as significant. 

## Results

The mean age of the patients was 42.4 ± 15.65 years and the mean BMI was 24.7 ± 6.33 kg/m^2^. The serum creatinine level of the patients was 2.20 ± 1.42 mg/dL, the stone size was 850 ± 121.43 mm², the mean operative time of the procedure was 125.76 ± 53.4 minutes, and the mean number of cell packs transfused was 1.10 ± 0.31 units. The mean preoperative Hb level was 12.88 ± 7.5 g/dL, the mean decrease in Hb level was 2.3 ± 1.98 g/dL, and the mean stone surface area was 5.9 ± 4.3 cm^2^. These descriptive statistics of all quantitative variables with their means and standard deviations are shown in Table 1.

**Table 1:** Analysis of quantitative variables BMI: body mass index, Hb: hemoglobin.

Variables	Mean		Standard deviation
Age (years)	42.4	15.65	
BMI (kg/m^2^)	24.7	6.33	
Serum creatinine level (mg/dL)	2.20	1.424	
Stone size (mm^2^)	850	121.43	
Operative time (min)	125.76	53.4	
Stone surface area (cm^2^)	5.9	4.3	
Number of pack cells (units)	1.10	0.31	
Pre-operative Hb level (g/dL)	12.88	7.5	
Decrease in Hb level (g/dL)	2.3	1.98	

Over 92 (70.2%) of the study subjects were male and 39 (29.8%) were female. Approximately one-third of the patients were female. 75 (57.25%) study subjects had a history of hypertension and 56 (42.75%) were normotensive patients. 78 (59.54%) of the study subjects were diabetic and 53 (40.46%) were non-diabetic patients. 75 (57.25%) study subjects had preoperative UTI and 56 (42.75%) did not have pre-operative UTI. Type of staghorn stone was also recorded; 49 (37.4%) of the patients had partial staghorn stone, 40 (30.5%) had complete staghorn stone, and 42 (32.1%) had multiple stones. 92 (70.2%) study subjects had chronic renal failure and 39 (29.8%) had a normal renal function. In socioeconomic status, it was found that most of the patients were from the middle class, i.e., 52 (39.69%), 31 (23.66%) were from the lower class, and 48 (36.65%) were from the upper class. Socioeconomic status did not affect the outcome. In the distribution of the number of tracts, 67 (51.14%) patients had single and the remaining had multiple tracts. Access calyx was also distributed, 28 (21.37%) patients had access from superior, 51 (38.93%) had access from medium, and the remaining 52 (39.69%) had access from the inferior calyx. 71 (54.2%) patients’ side of puncture was left and 60 (45.8%) patients’ side of puncture was right. This analysis of all qualitative variables is shown in Table 2.

**Table 2:** Analysis of qualitative variables UTI: urinary tract infection.

Variables		Frequency (n)	Percentages (%)
Gender	Male	92	70.2
	Female	39	29.8
Hypertension	Yes	75	57.25
	No	56	42.75
Diabetes mellitus	Yes	78	59.54
	No	53	40.46
Pre-operative UTI	Yes	75	57.25
	No	56	42.75
Staghorn stone	Partial staghorn stone	49	37.4
	Complete staghorn stone	40	30.5
	Multiple stones	42	32.1
Chronic renal failure	Yes	92	70.2
	No	39	29.8
Socioeconomic status	Lower class	31	23.66
	Middle class	52	39.69
	Upper class	48	36.65
Number of tract	Single	67	51.14
	Multiple	64	48.86
Access calyx	Superior	28	21.37
	Medium	51	38.93
	Inferior	52	39.69
Dilation method	Alken	38	29.0
	Amplatz	38	29.0
	Catheter-balloon	55	41.9
Side of puncture	Left	71	54.2
	Right	60	45.8

**Figure 1:**
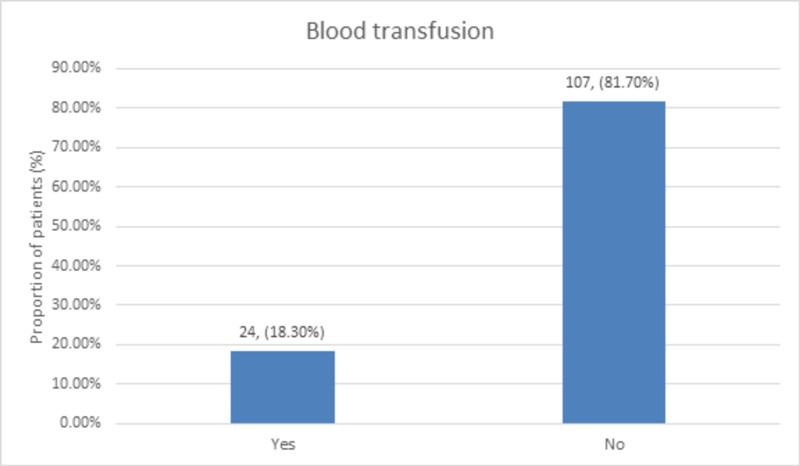
Distribution of blood transfusion

The blood transfusion was needed in 24 (18.3%) of the patients while 107 (81.7%) of the patients did not need a blood transfusion, as shown in Figure 1.

Stratification of age, BMI, size of the stone, operative time, preoperative Hb level, stone surface area (in cm^2^), hypertension, diabetes mellitus, preoperative UTI, gender, staghorn stone, chronic renal failure, socioeconomic status, number of tracts, access calyx, dilation method, and side of puncture with blood transfusion was performed. Gender, diabetes mellitus, stone size, preoperative Hb level, and operative time were significantly related to blood transfusion, as shown in Table 3.

**Table 3:** Stratification of blood transfusion with effect modifiers UTI: urinary tract infection, BMI: body mass index, Hb: hemoglobin. * p-value ≤0.05 is significant.

Variables		Blood transfusion		P-value
		Yes	No	
Gender	Male	9	83	0.003*
	Female	15	24	
Hypertension	Yes	10	65	0.111
	No	14	42	
Diabetes mellitus	Yes	7	71	0.01*
	No	17	36	
Preoperative UTI	Yes	18	57	0.067
	No	6	50	
Staghorn stone	Partial staghorn stone	9	40	0.78
	Complete staghorn stone	7	33	
	Multiple stones	8	34	
Chronic renal failure	Yes	15	77	0.45
	No	9	30	
Socioeconomic status	Lower class	6	25	0.93
	Middle class	10	42	
	Upper class	8	40	
Number of tract	Single	13	54	0.82
	Multiple	11	53	
Access calyx	Superior	5	23	0.42
	Medium	12	39	
	Inferior	7	45	
Dilation method	Alken	4	34	0.205
	Amplatz	10	28	
	Catheter-balloon	10	45	
Side of puncture	Left	14	57	0.82
	Right	10	50	
Age groups (years)	26-45	11	49	0.98
	46-70	13	58	
BMI (kg/m^2^)	<23	12	52	0.67
	>23	12	55	
Stone size (mm^2^)	<750	15	37	0.019*
	≥750	9	70	
Preoperative Hb level (g/dL)	<10	11	25	0.041*
	>10	13	82	
Operative time (minutes)	<125	8	67	0.012*
	≥125	16	40	

## Discussion

PNL, first discovered in 1976, has a high success rate of more than 90%. The probability of renal vascular injuries and grade IV renal injury requires evaluation [5]. Management of blood loss is through transfusion or transcatheter embolization in severe cases such as those resulting in massive hematuria and hemodynamic changes [9]. In one retrospective study, severe bleeding was found in 1% of the patients [10].

Staghorn stones have a high recurrence rate and require more interventions [11]. This was seen in a retrospective study of 2909 patients, where the staghorn stones were associated with an increased risk of bleeding requiring multiple tracts and excessive manipulation [9]. Additionally, a retrospective study of 177 staghorn calculi patients showed renal deterioration in 25% of the patients after surgery for staghorn calculi [12]. PNL is generally preferred over open surgery for staghorn and large-volume renal calculi. Open surgery has been associated with greater blood loss, while PNL is a minimally invasive procedure. However, complications still arise, with a complication rate of up to 83%, mostly including minor complications [4]. Renal bleeding is one of the most severe complications which requires angioembolization. Others include collecting system injuries, renal dysfunction, hypothermia, fluid overload, sepsis, urine leak, etc. [13]. Richstone et al. reported massive bleeding in 1.2% of their patients who eventually required angioembolization, with complete resolution of bleeding in 95% of the patients [14]. 

Several factors have been debated to be associated with increased bleeding risk, including staghorn calculi as mentioned above. PNL after percutaneous nephrostomy also increases the risk of hemorrhage requiring transfusion from between 0.5% and 4% to between 6% and 20%. Renal access is critical for predicting blood loss and should be compared to the blood transfusion rate. In a retrospective study including 1750 patients, the transfusion rate was highest with lower calyx access (12.6%) [15]. In another retrospective study, severe renal vascular injuries were found to be highest with upper calyx access at 4.6% compared to lower calyx access at 0.6%. In our study calyx access was not significantly associated with the frequency of blood transfusion. Instead, gender, diabetes mellitus, stone size, preoperative Hb level, and operative time were significantly associated. Other risk factors for increased bleeding risk include diabetes, staghorn stone, dilatation method, number of access, and stone size [16,17].

Taking a closer look into Table 3, the female gender predominates the gender ratio of the PNL procedure-related blood transfusion. In a local study, the transfusion rate was 18.7% higher than men. Their multivariate analysis showed staghorn stone, stone fragmentation with ultrasound, and chronic renal failure to be strongly associated with blood transfusion. In our study, the need for transfusion was 4.5% higher in women. Similarly, long operative time (p = 0.017) was significantly correlated with blood transfusion in a retrospective cross-sectional study [18]. More diabetic patients needed a blood transfusion in our study, making it a clinical factor associated with increased bleeding risk as seen in other studies as well [13]. However, another study comparing two cohorts of diabetic and non-diabetic groups, respectively, found no differences between major and minor bleeding in the patients [13].

There are some limitations to our study. Univariate and multivariate analysis can be done to further consolidate the comparison of the variables to blood transfusion. Our age group mostly includes older patients. A larger sample size with more variation in age groups is needed. Furthermore, conservative management and embolization groups can be created to further evaluate transfusion necessity as well as hospital stay and drop in hemoglobin after the procedure.

## Conclusions

PNL is a safe procedure for kidney stone removal, especially in the treatment of large stones. However, certain factors need to be controlled to remove the risk of severe bleeding. Access of the kidney should be appropriate to ensure that the kidney structure is minimally manipulated and the vasculature is not compromised. At the same time, operative time should not be too long. Female gender, stone size, diabetes mellitus, and preoperative Hb level may also predispose patients to more bleeding. Thus, bleeding risk can be reduced by controlling these factors.
